# FALCON2: compression-based metagenomic classification of ancient viruses

**DOI:** 10.1093/bioinformatics/btag155

**Published:** 2026-03-30

**Authors:** Luís L Marques, Armando J Pinho, Diogo Pratas

**Affiliations:** IEETA – Institute of Electronics and Informatics Engineering of Aveiro, University of Aveiro, Campus Universitário de Santiago, Aveiro, 3810-193, Portugal; DETI – Department of Electronics, Telecommunications and Informatics, University of Aveiro, Campus Universitário de Santiago, Aveiro, 3810-193, Portugal; LASI – Intelligent Systems Associate Laboratory, University of Aveiro, Campus Universitário de Santiago, Aveiro, 3810-193, Portugal; IEETA – Institute of Electronics and Informatics Engineering of Aveiro, University of Aveiro, Campus Universitário de Santiago, Aveiro, 3810-193, Portugal; DETI – Department of Electronics, Telecommunications and Informatics, University of Aveiro, Campus Universitário de Santiago, Aveiro, 3810-193, Portugal; LASI – Intelligent Systems Associate Laboratory, University of Aveiro, Campus Universitário de Santiago, Aveiro, 3810-193, Portugal; IEETA – Institute of Electronics and Informatics Engineering of Aveiro, University of Aveiro, Campus Universitário de Santiago, Aveiro, 3810-193, Portugal; DETI – Department of Electronics, Telecommunications and Informatics, University of Aveiro, Campus Universitário de Santiago, Aveiro, 3810-193, Portugal; LASI – Intelligent Systems Associate Laboratory, University of Aveiro, Campus Universitário de Santiago, Aveiro, 3810-193, Portugal; DoV – Department of Virology, University of Helsinki, Haartmaninkatu 3, Helsinki, 00014, Finland

## Abstract

**Motivation:**

Ancient DNA (aDNA) sequences present unique challenges for taxonomic classification due to extreme fragmentation (reads 20–100 bp), end-biased cytosine deamination, and high contamination rates. Conventional metagenomic classifiers based on exact *k*-mer matching or alignment lose discriminative power on such short and damaged reads, limiting the analysis of paleogenomic samples.

**Results:**

We present FALCON2, a compression-based metagenomic classifier that leverages position-aware finite-context models to maintain high accuracy on degraded viral ancient viruses. FALCON2 consolidates the capabilities of its predecessor, FALCON-meta, into a unified executable with enhanced features including model persistence, direct processing of compressed inputs, multiple file handling, and optional pre-filtering methodologies for contaminated samples. Under controlled benchmarking with database, taxonomy, and thread parity on simulated viral datasets, FALCON2 achieved an Area Under the Curve of Receiver Operating Characteristic (AUC-ROC) of 0.999, an Area Under Precision-Recall Curve (AUPRC) of 0.968, and an $F_{1}$-score of 0.918, substantially outperforming Centrifuge (AUPRC = 0.625), Kraken2 (AUPRC = 0.184), and CLARK-S (AUPRC = 0.013) on pooled micro-averaged metrics. FALCON2’s advantage is most pronounced on ultra-short reads (20–40 bp), where exact *k*-mers become sparse. FALCON2 pre-filtering at threshold 0.7 improved precision by 10 percentage points with negligible recall loss. FALCON2 runs on systems with 4–8 GB RAM for typical analyses.

**Availability and implementation:**

FALCON2 is freely available at https://github.com/cobilab/FALCON2 under GPL v3 license. Benchmarking data and scripts are archived at DOI: https://doi.org/10.5281/zenodo.17291214.

## 1 Introduction

Ancient DNA (aDNA) recovered from archaeologic, paleontologic and historical specimens yields key insights into extinct species, ancient microbiomes, and the evolutionary processes that shaped them (Reich et al. 2010, Enk et al. 2011).

However, aDNA sequences are typically characterized by extreme degradation: fragments are generally 20–100 bp in length, exhibit elevated cytosine deamination rates (C→T and G→A substitutions at read termini) and are often contaminated by modern and environmental DNA (Hofreiter et al. 2001, Briggs et al. 2007, Skoglund et al. 2014).

These properties compromise the reliability of conventional metagenomic classifiers, which rely on exact *k*-mer matches or long alignment anchors. In current practice, taxonomic classification of shotgun metagenomes follows two main paradigms: (i) exact or reduced *k*-mer methods with Lowest Common Ancestor (LCA) post-processing (e.g. Kraken2, Centrifuge, CLARK) (Ounit et al. 2015, Kim et al. 2016, Wood et al. 2019) and (ii) alignment-based LCA pipelines, such as MALT used in conjunction with MEGAN, typically relying on FM-index/BWT aligners (Herbig et al. 2017).

These pipelines are commonly paired with aDNA-aware mapping and processing frameworks that mitigate short, damaged reads (Schubert et al. 2012, 2014). They perform strongly on long, low-error reads but lose discriminative power as fragments shorten and post-mortem damage increases, the typical aDNA regime, because exact *k*-mers and long alignment anchors become sparse.

Marker-gene workflows (e.g. 16S/18S/ITS) and downstream functional imputation are powerful but target specific loci, making them less suitable for shotgun aDNA where fragmentation and damage are pervasive (Caporaso et al. 2010, Langille et al. 2013).

Compression-based similarity measures have long been used for alignment-free comparison and classification of biological sequences (Otu and Sayood 2003, Cilibrasi and Vitányi 2005, Ferragina et al. 2007, Pratas et al. 2018b, Munagala et al. 2022). In metagenomics, we introduced FALCON-meta, which avoids the need for long exact substrings by comparing relative compressibility between reads and references, retaining signal on ultra-short, damaged fragments (Pratas et al. 2018a).

FALCON-meta uses Finite-Context Models (FCMs) and Normalized Relative Similarity (NRS) to quantify sequence relationships without requiring exact substring matches. This approach demonstrated robustness to short and damaged reads, but was implemented as a fragmented codebase of multiple scripts, lacked model reuse capabilities, and did not address contamination filtering.

We present FALCON2, the successor to FALCON-meta, engineered as a unified, production-ready tool for ancient viral metagenomics. FALCON2 incorporates finite-context models, model persistence via serialization, native handling of compressed inputs (FASTA/FASTQ, gzip), multiple file processing, and optional integration of a lightweight compression-based pre-filter for removing contaminant reads. Benchmarking on synthetic viral datasets with factorial combinations of read length (20–100 bp), deamination rate (0%–30%), and sequencing depth (1–60×) shows that FALCON2 outperforms established nucleotide-based classifiers on short and damaged fragments while maintaining practical computational requirements.

## 2 Materials and methods

FALCON2 classifies metagenomic reads by computing NRS scores between query sequences and reference genomes (Pratas et al. 2018a). For each query sequence *x*, FALCON2 trains multiple FCMs on a reference sequence *y*, then encodes *x* using these reference-derived (frozen) models to obtain the compressed length C(x‖y).

**Figure 1:**
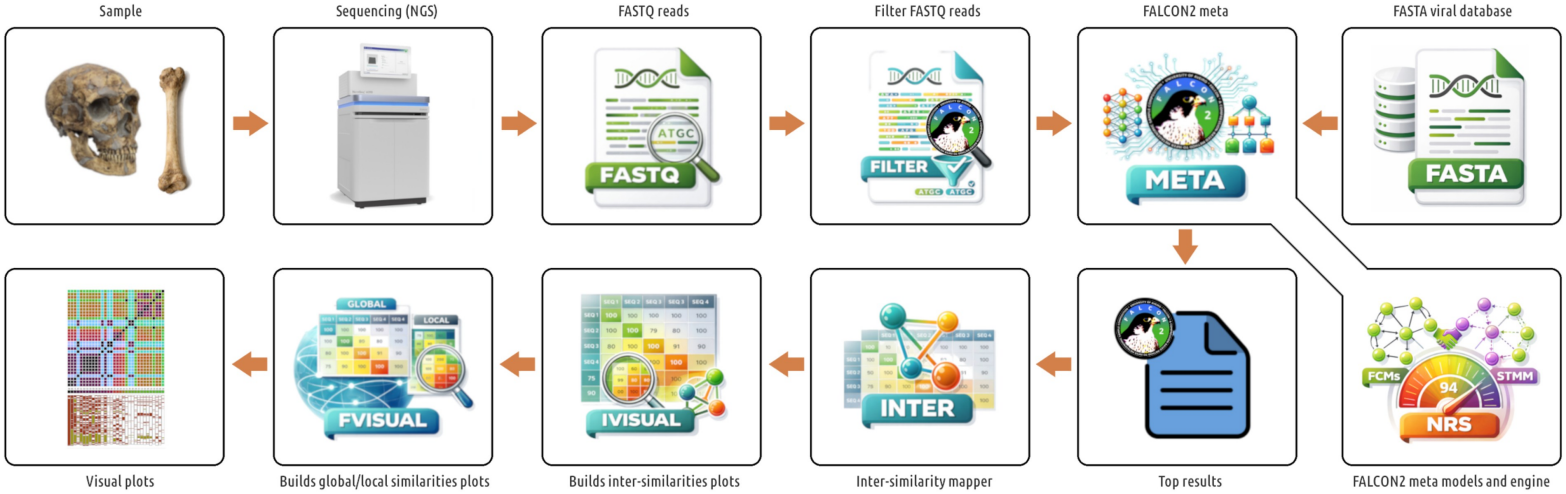
FALCON2 architecture and main functionalities: filter (FASTQ reads pre-filtering), meta (composition analysis using NRS with FCMs/STMMs), inter (inter-reference similarity computation), ivisual (similarity matrix heatmap rendering), and fvisual (global/local similarity profile visualization).

As depicted in Fig. 1, the FALCON2 architecture incorporates a method that uses a cooperative mixture of multi-order FCMs and substitution-tolerant Markov models (STMMs), which estimate symbol probabilities based on preceding context and allow for a degree of mismatch tolerance. The efficiency of relative compression depends on how well the reference-trained model collects and organizes information so that questions about the target can be answered with as few bits as possible.

This relative compression setup implies that the compressor cannot exploit intra-target redundancies, only information learned from the reference can reduce code length. Intuitively, if the reference describes the target well, the required relative information is small; if the target is not related, the encoder approaches the maximum number of bits.

Formally, the Relative Similarity (RS) is defined as $\text{RS}(x||y)=|x|{\;\text{log}\;}_{2}|\Theta |-C(x||y),$ and the normalization as


(1) $$\text{NRS}(x||y)=\frac{\text{RS}(x||y)}{\left|x|{\;\text{log}\;}_{2}|\Theta \right|}=1-\frac{C(x||y)}{\left|x|{\;\text{log}\;}_{2}|\Theta \right|},$$

where |x| is the size of the sequence and Θ is the size of the alphabet (4 for DNA). In read-level classification, FALCON2 assigns *x* to the reference from the database that produces the highest NRS (details in Section 1, available as supplementary data at *Bioinformatics* online). NRS is model-agnostic: any predictor for C(x‖y) applies. Neural lossless compressors may improve compression (Ma et al. 2025, Sun et al. 2025); a reference-trained neural model could thus replace FCMs. For ultra-short aDNA reads (20–100 bp), gains are uncertain and adaptive updates can confound C(x||y), so we keep CPU-native FCMs and flag neural/hybrid models as potential future works.

For composition profiling (the meta subcommand), we instead treat the sample as a bag/concatenation of reads *X* and compute NRS(y||X) for each reference *y* in the database, thus quantifying how well the sample explains each reference and allowing global composition estimates (Pratas et al. 2018a). For efficiency, the highest-scoring references (top-*K* by NRS) are cached and reused across computations.

FALCON-meta exposed functionality through several binaries: for global composition (FALCON), local-similarity filtering and visualization (FALCON-filter, FALCON-filter-visual), and inter-reference similarity (FALCON-inter, FALCON-inter-visual) (Pratas et al. 2018a). FALCON2 consolidates these into a single production-ready executable that presents equivalent capabilities as subcommands: meta for composition, filter/fvisual for segmentation and visualization of local profiles, and inter/ivisual for computing and rendering genome–genome similarity. The interface adds practical conventions, native streaming of gzip-compressed FASTA/FASTQ and colon-separated multi-file tokens for paired reads or multi-FASTA references, as well as model persistence and stricter reproducibility guaranties.

In routine use, composition runs as FALCON2 meta [options] READS_GROUP DB_GROUP, where each positional argument is a single colon-separated token (e.g. R1.fq.gz: R2.fq.gz for paired-end reads and ref1.fa:ref2.fa for multiple references). Inputs may be gzip compressed and are streamed directly, avoiding prior decompression. When local profiling is required, meta emits a profile in-process (e.g. with -Z -y profile.tsv), which is then segmented by filter and rendered by fvisual without format conversion. For database inspection and quality control, inter computes a genome-by-genome similarity matrix from the same multi-FASTA reference set, and ivisual produces a publication-ready heat map. Per-command help follows standard conventions and is available via FALCON2 <command> -h.

FALCON2 operates directly on data from sequencers, independently of coverage, and accepts both assembled references and non-assembled read sets. Although FALCON2 is designed for ancient viral metagenomics, it scales to large organismal databases (viral, bacterial, archaeal, fungal) as well as custom collections.

Trained FCMs can be serialized to .fcm files and reloaded in subsequent runs, enabling computational reuse in multi-sample analyses or when the reference database remains constant. Model persistence is implemented via the -S (save) and -L (load) flags, combined with -M to specify the model path. This functionality reduces steady-state inference time and ensures reproducibility across runs. For reproducibility, -T enables train-only runs and -I prints model metadata (tool version, reference snapshot hash, key parameters). Reloading (-L -M) enforces basic compatibility checks so that stale or mismatched models are rejected.

FALCON2 optionally integrates a compression-based pre-filter for contaminated samples. It computes approximate similarity scores between reads and a contaminant library (e.g. *E. coli*, human), retaining only reads with similarity below a configurable threshold τ (Sousa et al. 2024). Reads exceeding τ are excluded before FALCON2 classification, reducing computational load and improving precision. The pre-filter is activated with -mg, with threshold controlled by -mt (default 0.9; recommended 0.6–0.7 for aDNA).

Outputs are tabular files reporting NRS scores and taxonomic assignments for each read. Multi-threading is controlled via the -n parameter (default: all available cores). Internally, FALCON2 uses cache-aware hashing to memoize local probabilities and maintains a top-*K* cache of the highest NRS values across passes for speed. The compression depth parameter -l controls model fidelity; -l 47 was used in all benchmarking to maximize discrimination on short reads. The tool is freely available, under the GPLv3 license, at https://github.com/cobilab/FALCON2.

## 3 Benchmark

We benchmarked FALCON2 against Centrifuge (Kim et al. 2016), Kraken2 (Wood et al. 2019) and CLARK (Ounit et al. 2015) under strict parity conditions (details in Section 2, available as supplementary data at *Bioinformatics* online). All tools used identical reference databases (NCBI RefSeq-Viruses plus *E. coli* K-12 and human mitochondrial DNA as contaminants), as well as the same NCBI taxonomy snapshot, and fixed thread counts (*n* = 8). Synthetic datasets were generated via Gargammel (Renaud et al. 2017) (for aDNA fragmentation and deamination) and ART (Huang et al. 2012) (for sequencing errors), spanning combinations of read length (20, 40, 60, 80, 100 bp), deamination rate (0.0, 0.1, 0.2, 0.3), and sequencing depth (1, 5, 10, 20, 40, 60×). Ground truth was extracted from simulation metadata, and species-level precision, recall, $F_{1}$-score, AUPRC, and AUC-ROC were computed. AUPRC was prioritized over AUC-ROC due to class imbalance (Saito and Rehmsmeier 2015).

Table 1 summarizes pooled micro-averaged performance across all experimental conditions. Details are in Sections 3–9, available as supplementary data at *Bioinformatics* online. FALCON2 achieved the highest AUPRC (0.968), $F_{1}$-score (0.918), and AUC-ROC (0.999), substantially outperforming Centrifuge (AUPRC = 0.625, $F_{1}$ = 0.738), Kraken2 (AUPRC = 0.184, $F_{1}$ = 0.372), and CLARK (AUPRC = 0.013, $F_{1}$ = 0.103). The advantage was most pronounced at read length 20 bp, where FALCON2’s AUPRC exceeded Centrifuge by 0.34 and Kraken2 by 0.75 (Fig. 2, available as supplementary data at *Bioinformatics* online). At read length 100 bp with low deamination (0.0), performance differences narrowed (ΔAUPRC ≈ 0.06), consistent with the hypothesis that *k*-mer methods reassert efficiency on long, intact reads.

**Table 1:** Pooled micro-averaged performance across all conditions.^a^

Metric	FALCON2	Centrifuge	Kraken2	CLARK
Precision	**0.932**	0.753	0.508	0.116
$F_{1}$	**0.918**	0.738	0.372	0.103
AUC-ROC	**0.999**	0.914	0.666	0.548
AU-PRC	**0.968**	0.625	0.184	0.013
Time (min)	0.88	0.20	**0.04**	0.20
RAM (GB)	1.90	**0.07**	0.45	1.57

a Best values in bold. Time in minutes; RAM in GB. Runtime of CLARK is via CLARK-S.

Pre-filtering at threshold 0.7 increased precision from 0.85 to 0.95 while recall declined minimally from 0.90 to 0.87. The retained fraction k(τ) decreased from 0.70 to 0.30, indicating 70% of reads were filtered. An equivalence test confirmed that disabling filtering (τ=1.0) produced byte-identical outputs, validating orchestration integrity.

In summary, FALCON2’s compression-based approach maintains discriminative capacity on short and damaged aDNA reads where exact *k*-mer methods degrade. The integration of position-aware models, model persistence, and pre-filtering provides a robust, production-ready tool for ancient DNA metagenomics. The benchmarking framework enforces strict parity conditions and is fully reproducible via the archived scripts and data (DOI: 10.5281/zenodo.17291214).

FALCON2 exhibited higher runtime (median 0.88 min per sample) and memory usage (median 1.90 GB) than Kraken2 (0.04 min, 0.45 GB) and Centrifuge (0.20 min, 0.07 GB), reflecting the computational cost of FCM. However, absolute times remain practical for typical metagenomic workflows. On-disk footprint for the viral reference database was 75 MB (FALCON2), 45 MB (Centrifuge), and 255 MB (Kraken2). When using model persistence, build costs shift to a one-time training phase, and steady-state inference accelerates.

## 4 Conclusions

FALCON2 advances ancient viral metagenomic classification with compression-based models robust to fragmentation and deamination. Under controlled benchmarking, FALCON2 achieved superior AUPRC, $F_{1}$, and AUC-ROC compared to established classifiers, with the largest advantages on ultra-short reads (20–40 bp). The tool’s unified architecture, model reuse, and contamination filtering capabilities establish FALCON2 as an open-source solution for ancient viral metagenomic analysis. Although evaluated on ancient viral aDNA, FALCON2 is agnostic to organism type and is applicable to other highly degraded, contaminated short-read settings (e.g. eDNA or forensic DNA) given an appropriate reference database and, when relevant, a contaminant pre-filter library.

## Supplementary Material

btag155_Supplementary_Data
